# Composition, Mineral and Fatty Acid Profiles of Milk from Goats Fed with Different Proportions of Broccoli and Artichoke Plant By-Products

**DOI:** 10.3390/foods9060700

**Published:** 2020-06-01

**Authors:** Paula Monllor, Gema Romero, Alberto S. Atzori, Carlos A. Sandoval-Castro, Armín J. Ayala-Burgos, Amparo Roca, Esther Sendra, José Ramón Díaz

**Affiliations:** 1 Departamento de Tecnología Agroalimentaria, Universidad Miguel Hernández de Elche, 03312 Alicante, Spain; pmonllor@umh.es (P.M.); gemaromero@umh.es (G.R.); aroca@umh.es (A.R.); esther.sendra@umh.es (E.S.); 2 Dipartimento di Agraria, Università degli Studi di Sassari, 07100 Sassari, Italy; asatzori@uniss.it; 3 Facultad de Medicina Veterinaria y Zootecnia, Universidad Autónoma de Yucatán, Mérida 97100, Mexico; carlos.sandoval@correo.uady.mx (C.A.S.-C.); aayala@correo.uady.mx (A.J.A.-B.)

**Keywords:** fatty acid profile, mineral profile, CLA, milk yield, circular economy

## Abstract

In the Mediterranean region, artichoke and broccoli are major crops with a high amount of by-products that can be used as alternative feedstuffs for ruminants, lowering feed costs and enhancing milk sustainability while reducing the environmental impact of dairy production. However, nutritional quality of milk needs to be assured under these production conditions and an optimal inclusion ratio of silages should be determined. This work aimed to evaluate the effect of three inclusion levels (25%, 40%, and 60%) of these silages (artichoke plant, AP, and broccoli by-product, BB) in goat diets on milk yield, composition, and mineral and fatty profiles. Treatments with 60% inclusion of AP and BB presented the lowest milk yield. No differences were found on the milk mineral profile. Inclusion of AP in the animals’ diet improved the milk lipid profile from the point of view of human health (AI, TI) compared to BB due to a lower saturated fatty acid content (C12:0, C14:0, and C16:0) and a higher concentration of polyunsaturated fatty acids (PUFA), especially vaccenic acid (C18:1 trans11) and rumenic acid (CLA cis9, trans11), without any differences with the control treatment.

## 1. Introduction

Regarding milk consumption worldwide, cow’s milk occupies first place, followed by buffalo and thirdly, that of goat [1], which continues to increase [2] due to its high level of calcium, phosphorus, and animal protein. In addition, goat milk has been classified as a substitute for cow’s milk in those people who suffer from some type of allergy to this food [3]. Goat’s milk is a source of nutrients in the human diet due to its content of Se and polyunsaturated fatty acids (PUFA), such as vaccenic and rumenic acid or CLA [4,5], which can influence the prevention of certain types of cancers and cardiovascular diseases [6,7]. The literature contains many studies of how diet affects the performance and quality of ruminant milk. Hilali et al. [8] and Cappucci et al. [9] found that the inclusion of agro-industrial and olive by-products in ewes’ diets enhanced milk fatty acid profile, with no effects on performance and milk macro-composition. On the other hand, Schulz et al. [10] observed changes in milk fatty acid profile in cows fed with red clover silage in comparison with maize silage. Finally, Monllor et al. [11] showed slight differences in fat and protein levels of milk from goats fed with artichoke by-products and an increase of Selenium and polyunsaturared fatty acid contents. The inclusion of agricultural by-products in ruminant diets does not have to affect the sensory quality of dairy products. Such is the case in Caputo et al. Ref. [12], who did not observe differences in the aromatic profile of milk and dairy products from cows fed with destoned olive cake.

It is necessary to enhance the sustainability of milk production and reduce the impact of animal feeding. The use of local resources, especially if recovered from by-products, may significantly enhance milk sustainability. Artichoke (*Cynara scolymus* L.) and broccoli (*Brassica oleracea* var. Italica) crops generate large quantities of by-products. According to Food and Agriculture Organization of the United Nations (FAO) [13], 1,505,328 t of artichoke and 25,984,758 t of broccoli were harvested worldwide in 2017. The artichoke plant is a waste, mainly formed of stems and leaves, and some unharvested inflorescences are left in the field after harvest of inflorescences for human consumption. This by-product has traditionally been used by grazing small ruminants or collected and brought to dairy farms [14]. The yield of green fodder in this crop is 11.1 t/ha [15], which, taking into account FAO’s cultivated area data [13] (2017) worldwide (122,390 ha), would result in an annual production of more than 1,300,000 t of available artichoke plant. According to Ros et al. [16], 29.5% of harvested broccoli is composed of stems and inflorescences that are not suitable for human consumption. Broccoli by-product is considered, from the point of view of animal feed, more as a concentrate than as a forage, due to its low fibre content and high protein level [17].

Agri-food by-products, whether coming from stubbles left in the field or the canning industry, constitute a supply of alternative forage for livestock, allowing the use of local resources and reducing feed costs without damaging animal performance and productivity, as long as the rations that include these feeds are balanced. The use of these by-products can also be a solution to minimise residues produced by the agro-food industry and thus reduce removal costs and emissions of polluting gases caused by uncontrolled fermentation of these agricultural wastes. In addition, the use of agro-food by-products reduces the land and supplies dedicated to the development of livestock feed, thus aiding the circular economy. However, the strong seasonality and high water content of these feeds limits their systematic use in animal feeding. Through lactic fermentation, the silage is able to conserve perishable products so that cellular respiration is suppressed, protein and vitamin degradation is prevented, and clostridial fermentation is avoided [18], reaching levels of safety that do not endanger the health of animals and do not compromise the hygienic-sanitary quality of milk or derived products.

Previous studies have shown that these by-product silages have the proper fermentative and nutritional conditions to become part of sheep and goat diets [14,19,20]. The references found in the literature about the effect of consuming these silage by-products on milk quality and composition, as well as on the health status of animals, are scarce [21,22,23]. None of these studies have been conducted in dairy goats, except Muelas et al. and Monllor et al. [11,24], where the effect of up to 25% inclusion of silage artichoke plant on the technological aptitude of milk was studied.

With the previous background, it is hypothesised that these by-products may be incorporated into the diet of lactating goats without detriment to their milk yield and quality. Therefore, the objective of this experiment is to study the effect of the inclusion of by-product silages (artichoke plant and broccoli by-product) in the ration of goats on milk production, macro-composition, and quality and determine the optimum level of inclusion in the ration among the three levels tested (25%, 40%, and 60%), with the aim of assuring milk nutritional quality within an integrative approach of enhanced sustainability of milk production.

## 2. Materials and Methods

### 2.1. Animals and Facilities

The animals used in this experiment were Murciano-Granadina lactating goats housed in the experimental and teaching farm of the Miguel Hernández University, Spain, with access to outdoor yards (2.30 m^2^/animal), free access to water, and enough feeding space for all animals (at least 35 cm/animal and 1.50 m^2^/animal as total indoor space) with a straw bed. As usual in the region, the animals were milked once a day (Casse milking parlour, 2 × 12 × 12, GEA, Germany) and fed twice a day, at 8:00 a.m. and 2:00 p.m. This study was approved by the Ethical Committee of Experimentation of the Miguel Hernández University (code UMH.DTA.GRM.01.15).

### 2.2. Experimental Design

On the fourth month of lactation, 63 lactating goats were selected (41.2 ± 7.15 kg, 2.25 ± 0.80 kg/day, 5.39 ± 0.48 Log cell/mL). The animals were divided into seven homogeneous groups regarding body weight (BW), daily milk yield, and somatic cell count (SCC).

A short-term experiment was conducted to study the effect of inclusion in the diet of two by-product silages (artichoke plant, AP, and broccoli by-product, BB), of which their composition and fermentation quality are shown in Table 1. They were included at three levels each (25%, 40%, and 60%, expressed on a dry matter basis of the total ration); thus, seven rations were tested: 25%, 40%, and 60% of artichoke plant silage (AP25, AP40, and PAP60, respectively), the same percentages of broccoli by-product silage (BB25, BB40 and BB60), and a control diet (C), which represents the conventional ration used to feed dairy goats (alfalfa hay and a mixture of grains). Diets were formulated according to the recommendations of Fernandez et al. Ref. [25], an average amount of 2.23 kg DM/day was offered, and the seven rations were isoenergetic and isoproteic. Table 2 shows the ingredient proportion and the chemical composition of each diet. Once the pre-experimental sampling was performed, the experiment lasted 4 weeks. In the first two weeks, each group of animals adapted to their diet. In the next two weeks, data on feed consumption, milk yield, and body weight were recorded and blood and milk samples from animals were collected weekly for subsequent laboratory analyses. Bulk milk samples were collected weekly and used to determine mineral and fatty acid profile concentrations.

### 2.3. Analysed Variables

The body weight of the animals (BW, kg) was recorded by weighing them on a scale (±100 g, APC, Baxtran, Vilamalla, Spain). The feed consumption was measured twice a week and calculated by the average of the difference of the feed offered and refused on dry matter basis. The chemical composition of the silages and diets was analysed as previously described by Monllor et al. [11]. Dry matter (DM, g/kg; method 930.5), organic matter (OM, g/kg DM; method 942.05), ether extract (EE, g/kg DM; method 920.39), crude protein (CP, g/kg DM; method 984.13), and crude fibre (CF; g/kg DM; method 962.09) were determined following AOAC [27] procedures. Neutral detergent fibre (NDF, g/kg DM), acid detergent fibre (ADF, g/kg DM), and acid detergent lignin (ADL, g/kg DM) were analysed according to Van Soest et al. [28]. Total polyphenol content (TP, g/kg DM) was measured by the Folin-Ciocalteu method [29]. Volatile fatty acids (VFA, g/kg DM) (acetic, propionic, and butyric acid, also including lactic acid and ethanol) were determined by HPLC liquid chromatography (Agilent 1200, Santa Clara, CA, USA and Supelcogel C-610H column: 30 cm × 7.8 mm ID, Saint Louis, MO, USA), by Feng-Xia et al. [30] methodology. Apparent in vitro dry matter digestibility (IVDMD, g/kg DM) was measured according to Menke and Steingass [31]. Fatty acid profile analysis in diets was performed by direct methylation on the lyophilised samples, without prior extraction of the fat, according to Kramer et al. [32] and were identified by a gas chromatograph (GC-17A Shimadzu, Kyoto, Japan) coupled with a flame ionisation detector (FID) equipped with a capillary column (CP Sil 88 100 m × 0.25 mm internal diameter and 0.20 µm internal coverage, Agilent, Santa Clara, CA, USA). A mixture of fatty acid methylated esters (FAME;18912-1AMP, Sigma-Aldrich, Saint Louis, MO, USA) was used for identification of the fatty acids of the samples.

Dietary and milk minerals (Na, Mg, K, Ca, P, S, Se, Zn, Cu, Fe, and Mn) were determined by carrying out a previous digestion of the samples, according to González Arrojo et al. [6]. Microwave (MW) digestion unit Ethos Easy, Milestone (Milestone, Srl, Sorisole, Italy) equipped with a rotor for 10 TFM (chemically modified PTFE) vessels was used for sample mineralisation. The microwave program consisted of four phases (i) 5 min at 1000 W at temperatures from 100 to 60 °C; (ii) 10 min at 1000 W from 165 to 80 °C; (iii) 5 min at 1000 W from 180 to 120 °C; and, (iv) 5 min at 700 W from 180 to 120 °C. The ICP-MS (inductively coupled plasma mass spectrometry) instrument used in this study was an Agilent 7700× Octopole Reaction System (ORS) (Agilent Technologies, Tokyo, Japan). The ICP-MS operating conditions were optimised for the simultaneous determinations of all elements. ICP-MS standard solutions were prepared daily by appropriate dilution of stock standard 1000 mg/L for each element in 2% *v*/*v* Suprapur HNO3. An appropriate internal standard was also required for each analyte to correct physical and/or matrix interferences in ICP-MS.

The milk yield (kg/day) of every goat was determined during milking using a Lactocorder^®^ device (Lactocorder, Balgach, Switzerland). This device collected a representative sample of 100 mL of milk at every milking of each animal for subsequent analysis. The macro-composition of milk (fat, protein, true protein, casein, whey protein, lactose, total solids, TS; non-fat total solids, NFTS; useful dry matter content, UDM, and ash; %) and urea content (mg/L) was determined by medium infrared spectroscopy equipment (MilkoScan™ FT2, Foss, Hillerød, Denmark). The SCC (10^3^ cell/mL) was analysed by an electronic fluoro-optical method (DCC, DeLaval, Tumba, Sweden). Fat corrected milk yield (FCM) was calculated according to Gravert equation [33]: FCM (3.5%) = 0.433 × milk yield (kg/day) + 16.218 × fat milk yield (kg/day). Milk fatty acids were extracted by the Folch procedure, with some variations collected in Romeu-Nadal et al. [34] and were methylated following the Nudda et al. [35] method. The equipment, column, and FAME mix used for the identification of peaks of milk fatty acid profile were the same as for the diets. Atherogenicity index (AI) and thrombogenicity index (TI) were calculated according to Ulbricht and Southgate [36]. These indices provide important information because AI is related with the ability of lipids’ adhesion to immunological and circulatory system cells and TI indicates the tendency to form clots in blood vessels [8]. Desaturase indices (DI) for C14:0, C16:0, and C18:0 were calculated according to Lock and Garnsworthy [37].

In order to assess the effect of the diets on goats’ metabolism, blood samples were analysed. The same day as the milk sampling was performed, the fasting animals were bled and samples were collected for glucose, urea, and β-hydroxybutyrate (BHB) analysis. Blood samples were analysed with a glucose oxidase/peroxidase kit (Ref. 11503, Biosystems, Barcelona, Spain) for glucose (mg/dL), with a kinetic method (GN 10125, Gernon, Sant Joan Despí, Spain) for urea (mg/dL), and for the BHB (mmol/L), the Ranbut D-3-Hydroxybutyrate kit (RB 1007, Randox, Crumlin, UK) was used.

### 2.4. Calculations and Statistical Analysis

The SCC data were transformed into log_10_ scores before statistical analysis (LSCC).

BW, milk yield and macro-composition, SCC, and plasmatic profile data were performed using SAS GLIMMIX (SAS Institute Inc., Cary, NC, USA) with repeated measures, introducing the covariate of the data obtained in the pre-experimental sampling into the model and considering DIET, SAMPLING, and interaction DIET × SAMPLING as fixed effects, according to the following equation:

*Y* = *µ* + *Di* + *Sj* + *DixSj* + *covY0* + *Ak* + *e,*

where *Y* is the dependent variable, *µ* is the intercept, Di is the fixed effect of the diet (*i* = C, AP25, AP40, AP60, BB25, BB40, BB60), *Sj* is the fixed effect of sampling (*j* = 1, 2, 3), *DixSj* is the interaction of diet with sampling, *covY0* is the effect of the value of *Y* in sampling 0, *Ak* is the random effect of the animal, and *e* is the residual error. The covariance model with a lower value of the Akaike criterion (lower AIC and BIC) was used for each variable.

Milk mineral and fatty acid profile data were analysed using SAS GLM (SAS Institute Inc., Cary, NC, USA), introducing the covariate of the data obtained in the pre-experimental sampling into the model and considering DIET as a fixed effect. The level of acceptance for significance was 0.05.

## 3. Results

### 3.1. Diet Effects on Body Weight and Feed Consumption

Body weight is an indicator of the health status of the animal and optimising the inclusion of by-products involves assuring the proper health status of the goats. The treatments with the highest by-product inclusion showed a lower BW (40.2 and 38.7 kg in AP60 and BB60, respectively), while with the inclusion of 25% and 40%, no differences were observed compared to C (42.9 kg, Table 3). Sampling and interaction Treatment × Sampling also had a significant effect on BW as an increase (*p* < 0.001) was observed in sampling 2 in treatments with 40% of by-product (+1.9 and +2.4 kg in BB40 and AP40, respectively) and then in sampling 3, they descended again. Diets were offered in a similar amount but the goats in the different treatments showed different consumptions, with group C showing the highest (2.21 kg DM/day), whereas the lowest consumption was observed in groups BB40 (1.38 kg DM/day) and BB60 (1.27 kg DM/day) compared to the other treatments, which showed intermediate consumption (AP25: 1.52, AP40: 1.54, AP60: 1.57, and BB25: 1.65 kg DM/day).

### 3.2. Milk Yield, Macro-Composition, and SCC

A decrease in milk yield was observed as the percentage of inclusion of by-products increased (Table 3). C, AP25, and AP40 were the treatments with the highest milk daily yield (2.24, 2.15, and 2.14 kg/day, respectively; *p* < 0.001); BB60 was associated with the lowest yield (1.66 kg/day). A tendency to decrease FCM was also observed as the percentage of inclusion of the by-product in the diet increased. The highest yield was obtained in AP25, even without significant differences compared to C or other AP treatments; BB25 and BB40 did not show significant differences compared to C, AP40, and AP60, whereas BB60 showed the lowest value. The interaction among sampling and treatments was significant as the milk yield and FCM were only significantly reduced in AP25 and AP60 during the experiment, but remained stable in the rest of the treatments.

The diet had no significant effect on LSCC. An increase of + 0.28 Log cells/mL (*p* < 0.01) was observed in AP25 between samplings 2 and 3, so that sampling and interaction with treatment were significant.

As for the macro-composition of the milk shown in Table 3, the diet only had significant effects on fat (but also affected UDM and TS) and urea (Table 3). BB60 was the one with the highest fat value and T was the lowest. The significant interaction of the treatment with the sampling in fat, UDM, TS, whey protein, and lactose was due to specific increases or decreases in sampling 2 in AP40, which returned to similar values to the previous ones at sampling 3. Both the casein content of milk and NFTS were reduced in all treatments during the experiment (*p* < 0.001). The ash content increased 0.134 percentage units in AP25 at the end of the experiment, remaining stable in the rest of the treatments. Regarding the milk urea content, AP60 was the treatment that presented the highest level (641 mg/dL; *p* < 0.01) and BB60 the lowest (542 mg/dL).

### 3.3. Milk Mineral Content

Milk mineral profile is shown in Table 4. Only significant differences in the Mn concentration due to dietary treatment were observed, although of small magnitude. AP40 was the treatment that presented the highest level of Mn (0.233 mg/kg DM; *p* < 0.05), followed by BB25 (0.222 mg/kg DM), whereas BB40 was the treatment showing the lowest value (0.185 mg/kg DM). These differences between treatments are not considered biologically relevant because the greatest of them, which was between AP40 and BB40, was only 0.048 mg/kg DM.

### 3.4. Milk Fatty Acid Profile

Regarding the milk fatty acid profile (Table 5), some significant variations were observed, although they were quantitatively limited. Regarding the content of vaccenic acid (C18:1t11), it was observed that this was higher (*p* < 0.001) in the AP treatments, without differences compared to C. There was a higher concentration of linoleic acid (C18:2n6) in AP60 (2.53%; *p* < 0.001); however, it was at C where a higher level of other C18: 2 isomers was observed. An increase (*p* < 0.001) of α-linolenic acid (C18:3n3) was observed as the level of AP inclusion in the ration was higher and AP60 presented a higher level (0.242%). AP treatments were also those with the highest content (*p* < 0.01) in rumenic acid (CLA c9, t11), although subsequently no significant differences were found in the sum of isomers of CLA (conjugated linoleic acid) between treatments, except of BB60, of which their content was the smallest of all. Table 6 shows that as the percentage of AP inclusion increased, so did the PUFA content, and AP60 was the treatment with the highest content (*p* < 0.001) compared to all the BB treatments, without differences from C or the rest of the AP treatments. AP60 presented the highest levels (*p* < 0.001) of n3 (0.275%) and n6 (2.79%) fatty acids, the latter without differences compared to C or the other AP treatments. It also achieved the lowest (*p* < 0.001) ratio n6/n3 obtained together with BB60 (10.3 and 12.3, respectively). Regarding the lipid quality indices related to human health (AI and TI), AP40 and AP60 were the ones with the lowest value (*p* < 0.001) and therefore, were healthier. Regarding the desaturation indices of the myristic (DI14), palmitic (DI16), and stearic (DI18) fatty acids, the differences found between treatments were of small magnitude. BB60 was the one with the highest value in DI14 and DI18 (0.012% and 2.08%, respectively; *p* < 0.001) and AP60 presented a higher value of DI16 (0.061%; *p* < 0.01).

### 3.5. Plasma Metabolic Profile

Regarding the plasma metabolic profile (Table 7), it was observed that the greater the inclusion of BB in the diet, the higher the glucose level (49.5 and 50.0 mg/dL in BB40 and BB60; *p* < 0.001), although the differences were of small magnitude (42.5 mg/dL in BB25). Regarding urea, C and AP had a higher content (*p* < 0.001), while the BB treatments obtained lower levels and BB60 showed the lowest (33.2 mg/dL). The level of BHB was higher in treatments that included less by-product, such as AP25, AP40, and BB25, while it was lower in treatments that included more BB (0.299 and 0.304 mmol/L in BB40 and BB60, respectively; *p* < 0.001). There was significant interaction of treatment with sampling in the three variables due to the different behaviour throughout the experiment between treatments: Glucose increased (*p* < 0.001) with the progress of the experiment in all treatments except BB60; blood urea was reduced (*p* < 0.001) at sampling 2 in BB25 and BB40 and increased at sampling 3 in BB25, BB40, and BB60; BHB increased (*p* < 0.01) at the end of the experiment in BB25, BB60, and AP60, while in C, BB40, AP25, and AP40 remained stable.

## 4. Discussion

### 4.1. Diet Effects on Body Weight and Feed Consumption

One of the factors that affects the total volume of the diet and its consumption by livestock is the moisture content, as Jackson and Forbes [38] pointed out. This effect is especially important in the short term as herbivores are able to progressively modify the volume of the rumen to increase the speed of transit of the digesta [39], so in the long term, this effect would have less influence. In this experiment, carried out in the short term, diet C was the one presenting the highest DM content and feed consumption (2.21 kg DM/day). On the contrary, diets BB40 and BB60 contained a greater amount of water and were bulkier and presented less consumption. In addition, diets with silage showed higher concentrations of VFA and other substances resulting from fermentation. The presence of propionic acid in BB60 (4.79 g/kg DM), as well as a higher concentration of ammonia N in both BB40 and BB60, also occurred in treatments with lower consumption due to the depressing effect on feed consumption demonstrated by Baumont [40]. The feed consumption of the BB treatments was superior to those found by Meneses [41] (0.508 kg DM/day) in Murciano-Granadina castrated males, whose ration incorporated 55% of BB silage. All BW values were normal for the Murciano-Granadina breed [42,43]. The greatest reduction in BW was in BB60, as well as the greatest reduction in feed consumption (1.27 kg DM/day and 38.7 kg).

### 4.2. Milk Yield, Macro-Composition, and SCC

The treatments that presented a higher feed consumption were those that had a higher milk yield. The values obtained are similar to the yield obtained with the equation proposed by León et al. Ref. [44] for the modelling of the Murciano-Granadina lactation curve, which stands at 1.93 kg/day between the fourth and fifth lactation months, which is where the animals used in this experiment were located. The highest percentage of fat in BB60 (4.59%) was probably due to a concentration effect (being the treatment with the lowest yield) and its highest content in acetic acid (37.8 g/kg DM, triple the rest) in the diet, which is an extra-lipogenic nutrient precursor of fat synthesis. Van Knegsel et al. [45] observed similar effects in dairy cows when part of the corn in the diet was replaced by beet pulp. Due to a higher fat concentration in BB60, UDM and TS also reached the highest values in this treatment (8.03% and 12.9%, respectively), exceeding C by almost a percentage point. The urea level of all treatments was found to be within the optimal range for goats recommended by the Interprofessional Dairy Laboratory of Castilla-La Mancha (LILCAM), which is between 500 and 700 mg/L. The differences found in the milk urea content can be explained by the different levels of feed consumption of the treatments. BB60 presented less feed consumption, in particular refusing part of the offered BB, which probably induced lower total protein intake and lower levels of milk urea, as Jimeno et al. [46] noticed.

### 4.3. Milk Mineral Content

The macromineral values correspond to those found by Mellado and García [47] in goat crossings. The composition of the diet of animals largely determines the concentrations of minerals in milk [48]. As there were no large differences in the content of the different minerals in the diets, no significant differences were subsequently observed in the milk of the different treatments, which is important for the technological aptitude of the milk, given the relevance of Ca and P in the setting and development of the microstructure of cheese [49], the main destination of goat’s milk. Only the Mn had a higher concentration in AP40 (0.233 mg/kg DM), although with such tight differences that they are not biologically relevant.

### 4.4. Milk Fatty Acid Profile

The milk of animals fed with AP60 had a higher content of n3 fatty acids, which caused a lower n6/n3 ratio, which is positive for the prevention of coronary and cardiovascular diseases [50]. On the other hand, C, AP25, AP40, and AP60, of which their diets had the highest levels of PUFA, were the treatments with milk richest in vaccenic, rumenic, and PUFA, as reported by Collomb et al. [51], who observed differences in the PUFA and vaccenic content in the milk of cows fed with high mountain pastures and in lowland plains because the plants that made up the mountain meadows had a higher concentration of PUFA.

Another factor that could influence the increase of PUFA in AP treatments was the slightly higher content of total polyphenols (TP) in the diet, although lower than that of BB60. However, the lower feed consumption of BB60 could mean that the total TP intake does not reach those of the AP treatments. Several studies have demonstrated the inhibitory action of dietary polyphenols on ruminal biohydrogenation of PUFA, without detrimental effects on milk yield and composition, due to interference with microbial flora [52,53,54,55]. These effects have also been observed in sheep with small amounts in the diet of by-products rich in TP [56,57]. Cappucci et al. Ref. [9] also observed how after increasing the TP content of the diet of Comisana sheep by including different levels of olive by-product, the concentration of linoleic (C18:2n6) and α-linolenic (C18:3n3) in milk was increased.

As a result of a lower milk content of C12:0, C14:0, C16:0, and C18:0, AP40 and AP60 had the lowest levels of AI and TI, so the milk of these animals would be of higher quality in terms of human health [42]. The values obtained from AI in all the treatments of this study are below those found by Molina-Alcaide et al. Ref. [42] in Murciano goats fed with conventional ration supplemented with feed blocks of olive by-products. The desaturation indices obtained in this experiment are similar to those provided by Baldin et al. Ref. [58] in a study in goats that received a dietary CLA supplement.

### 4.5. Plasma Metabolic Profile

Despite the differences found in the metabolic profile of the different treatments, glucose, urea, and BHB levels remained within the ranges considered optimal for goats [59], except for the urea value in BB60, which was slightly lower. As Friggens et al. [60] observed in goats’ performance, the level of BHB was generally low and particularly in those treatments showing lower feed consumption (BB40 and BB60) because goats, as lactating animals, adapt their milk yield to the level of feed intake, as seen in Table 3. This reduces the metabolic load and allows them to maintain adequate body reserves turnover. Due to the strong relationship between plasma and milk urea content [61], the lower levels of blood urea were found in the same treatments with the lowest values of milk urea.

## 5. Conclusions

The findings of this study highlighted that a threshold level of AP or BB inclusion in dairy goat diets, without negative effects on milk yield, composition, mineral and fatty acid profile, as well as metabolic status of the animals, would be 40% of the dietary dry matter.

The inclusion of artichoke plant and broccoli by-product silages in high doses (60%) caused lower feed consumption and lower milk yield. Inclusion at 60% of AP and BB increased the milk TS, although not enough to compensate for the reduced yield, resulting in lower FCM in the case of BB60. No differences were found regarding the milk mineral profile. Inclusion of the artichoke plant silage in the animals’ diet improved the milk lipid profile from the point of view of human health (AI, TI) compared to broccoli silage, due to a lower SFA content (C12:0, C14:0, and C16:0) and a higher concentration of PUFA, especially vaccenic acid (C18:1 trans11) and rumenic acid (CLA cis9, trans11), without any differences compared to the control treatment. Regarding sanitary status, the plasma metabolic profile in broccoli treatments reflects that goats ate grains and alfalfa, whereas broccoli was the last ingredient, impairing its consumption, especially at the high concentration (60%). In addition, the diets that included 60% of by-product silages caused a reduction in BW.

## Figures and Tables

**Table 1:** Chemical composition (g/kg DM) and fermentation quality (g/kg DM) of silages included in experimental diets.

Item	BB	AP
**Chemical Composition**		
DM (g/kg of FM, as fed)	154	258
OM	821	828
CP	174	78.1
CF	214	296
NDF	430	571
ADF	326	374
ADL	63.4	108
EE	32.1	34.6
TP	6.73	4.96
**VFA and Fermentative Metabolites**		
Lactate	30.8	17.0
Acetate	117	35.2
Propionate	14.6	n.d.
Butyrate	3.80	8.56
Ethanol	14.6	3.25
Ammonia N	1.65	0.149

BB: Broccoli by-product silage; AP: Artichoke plant silage; DM: Dry matter; FM: Fresh matter; OM: Organic matter; CP: Crude protein; CF: Crude fibre; NDF: Neutral detergent fibre; ADF: Acid detergent fibre; ADL: Acid detergent lignin; EE: Ether extract; TP: Total polyphenols; VFA: Volatile fatty acids; n.d.: Not detected.

**Table 2:** Ingredients of experimental diets and their nutritional value.

Item	Diets						
	C	AP25	AP40	AP60	BB25	BB40	BB60
**Ingredients (g/100 g DM)**							
Alfalfa hay	38.0	14.7	-	-	13.5	8.50	4.60
Oat	16.0	15.0	13.0	8.0	35.0	26.5	26.6
Barley	9.50	9.00	8.00	4.51	5.50	3.72	1.23
Corn	9.08	8.43	8.00	4.35	5.16	3.60	1.19
Dried sugar beet pulp	7.36	7.00	6.50	3.53	4.18	3.00	0.960
Sunflower meal	3.36	3.12	3.00	1.61	2.00	1.33	0.440
Peas	2.50	2.32	2.09	1.20	1.42	0.990	0.330
Cottonseed	2.50	2.32	2.09	1.20	1.42	0.990	0.330
Soybean meal 44%	4.00	6.00	10.0	12.0	2.00	2.00	1.00
Corn DDGS	3.00	3.00	2.50	1.38	2.00	1.14	0.380
Sunflower seeds	2.00	1.74	2.40	1.00	1.07	0.740	0.250
Beans	1.25	1.16	1.05	0.600	1.00	0.500	0.160
Wheat	1.00	0.770	1.00	0.400	0.470	0.330	0.110
Soy hulls	0.420	0.390	0.350	0.200	0.240	0.160	0.050
Silage	-	25.0	40.0	60.0	25.0	40.0	60.0
kg DM offered/day/animal	2.24	2.26	2.20	2.30	2.22	2.21	2.20
**Chemical Composition**							
DM (g/kg FM)	893	554	448	361	438	334	254
g/kg DM							
OM	935	915	901	884	916	904	885
CP	162	160	163	157	162	165	169
CF	195	202	196	237	180	180	183
NDF	376	391	382	432	359	355	353
ADF	243	248	239	281	225	226	231
ADL	56.5	55.1	49.5	55.2	48.0	47.0	46.7
EE	41.9	36.5	35.1	30.5	41.3	38.5	34.7
TP	3.87	4.18	5.42	5.34	4.60	5.42	6.68
IVDMD	715	715	710	665	780	747	757
^1^ME (Mcal/kg DM)	2.37	2.30	2.29	2.19	2.39	2.36	2.29
**VFA and Fermentative Metabolites (g/kg DM)**							
Lactate	n.d.	14.2	23.2	24.5	33.1	41.2	56.0
Acetate	n.d.	4.91	6.04	11.9	15.1	11.0	37.8
Propionate	n.d.	n.d.	n.d.	n.d.	2.63	n.d.	4.79
Butyrate	n.d.	n.d.	n.d.	n.d.	n.d.	n.d.	n.d.
Ethanol	n.d.	1.50	1.80	1.69	9.64	12.5	23.2
Ammonia N	0.166	0.628	0.741	1.01	3.99	4.26	7.73
**Fatty Acids Profile (g/100 g Total Fatty Acids)**							
C6:0	0.061	0.109	0.485	0.352	0.059	0.025	0.498
C12:0	0.183	0.286	0.151	0.050	0.242	0.328	0.146
C14:0	0.440	0.502	0.413	0.357	0.542	0.539	0.465
C16:0	17.2	18.1	18.3	17.3	19.8	17.7	21.2
C16:1 c9	0.300	0.348	0.369	0.364	0.374	0.312	0.592
C18:0	3.25	3.08	2.93	3.63	2.96	3.34	2.76
C18:1 c9	26.4	25.1	22.8	31.3	30.1	34.3	21.9
C18:1 c11	1.06	1.11	1.33	1.12	2.00	2.23	3.74
C18:2n6	44.0	42.0	40.5	32.3	35.5	29.4	29.4
C18:3n3	4.07	4.79	6.75	6.43	5.79	8.18	13.0
C20:0	0.463	0.757	0.884	1.19	0.493	0.679	0.838
C20:1n9	0.323	0.408	0.300	0.336	0.464	0.386	0.423
C22:0	0.457	0.546	0.519	0.960	0.393	0.784	0.640
C24:0	0.336	0.493	0.392	0.411	0.365	0.600	0.652
SFA	23.3	24.7	26.4	26.8	25.5	24.6	29.5
MUFA	28.2	27.6	26.1	33.7	33.0	37.5	27.5
PUFA	48.7	48.3	47.7	40.0	41.5	38.1	43.2
**Mineral Profile**							
Na (g/kg DM)	2.89	5.83	7.34	12.1	2.37	5.28	5.09
Mg (g/kg DM)	2.66	3.24	3.05	3.63	2.06	2.52	2.43
K (g/kg DM)	13.5	14.3	14.1	17.8	17.8	19.4	30.1
Ca (g/kg DM)	5.90	10.8	11.2	17.0	5.62	8.91	7.49
P (g/kg DM)	2.76	4.09	3.69	3.56	3.40	3.61	4.18
S (g/kg DM)	2.89	3.45	3.06	3.78	3.40	4.27	6.58
Se (mg/kg DM)	0.198	0.190	0.150	0.243	0.183	0.135	0.167
Zn (mg/kg DM)	49.4	44.2	41.3	34.1	43.6	42.5	36.9
Cu (mg/kg DM)	6.15	6.42	5.83	6.76	5.68	4.67	5.41
Fe (mg/kg DM)	129	414	287	460	175	161	235
Mn (mg/kg DM)	42.1	47.7	44.2	54.0	38.5	34.6	35.7

C: Control diet; AP: Diet that includes artichoke plant silage; BB: Diet that includes broccoli by-product silage; DM: Dry matter; FM: Fresh matter; OM: Organic matter; CP: Crude protein; CF: Crude fibre; NDF: Neutral detergent fibre; ADF: Acid detergent fibre; ADL: Acid detergent lignin; EE: Ether extract; TP: Total polyphenols; IVDMD: In vitro dry matter digestibility; ME: Metabolisable energy; VFA: volatile fatty acids; SFA: Saturated fatty acids; MUFA: Monounsaturated fatty acids; PUFA: Polyunsaturated fatty acids, n.d.: Not detected ^1^[26].

**Table 3:** Body weight, milk yield, and composition and SCC, according to the effects considered.

Variable	Diets								Significance		
	C	AP25	AP40	AP60	BB25	BB40	BB60	SEM	Diet	Sampling	Diet x Sampling
BW (kg)	42.9 a	41.6 ab	42.2 a	40.2 bc	41.9 ab	41.9 ab	38.7 c	0.69	***	***	***
Milk yield (kg/day)	2.24 a	2.15 ab	2.14 abc	1.92 bcd	1.90 cde	1.76 de	1.66 e	0.090	***	**	***
LSCC (Log_10_ cell/mL)	5.53	5.67	5.58	5.68	5.54	5.82	5.68	0.109	n.s	**	**
FCM (3.5%; kg/day)	2.31 ab	2.42 a	2.26 ab	2.17 abc	2.03 bc	2.00 bc	1.88 c	0.120	**	**	*
Fat (%)	3.76 b	4.25 ab	4.06 ab	4.29 ab	4.02 ab	4.25 ab	4.58 a	0.218	**	n.s.	*
Protein (%)	3.39	3.42	3.52	3.39	3.34	3.34	3.42	0.088	n.s.	n.s.	n.s.
UDM (%)	7.15 b	7.68 ab	7.59 ab	7.68 ab	7.36 ab	7.61 ab	8.01 a	0.275	*	n.s.	*
True protein (%)	3.16	3.18	3.27	3.15	3.11	3.11	3.18	0.078	n.s.	n.s.	n.s.
Casein (%)	2.68	2.69	2.76	2.66	2.65	2.65	2.72	0.061	n.s.	***	n.s.
Whey protein (%)	0.470	0.484	0.507	0.491	0.456	0.465	0.474	0.024	n.s.	***	**
Lactose (%)	4.25	4.16	4.20	4.16	4.23	4.20	4.18	0.045	n.s.	***	**
TS (%)	12.0 b	12.5 ab	12.4 ab	12.4 ab	12.2 ab	12.4 ab	12.9 a	0.28	*	*	*
NFTS (%)	8.75	8.67	8.81	8.63	8.70	8.67	8.75	0.084	n.s.	***	n.s.
Ash (%)	0.639	0.615	0.648	0.625	0.638	0.627	0.652	0.024	n.s.	n.s.	*
Milk urea (mg/L)	617 ab	587 abc	591 abc	641 a	558 bc	588 abc	542 c	23.0	**	n.s.	n.s.

C: Control diet; AP: Diet that includes artichoke plant silage; BB: Diet that includes broccoli by-product silage; 25, 40, and 60 inclusion level of by-product silage on dry matter basis %; SEM: Standard error mean; BW: Body weight; LSCC: Log_10_ somatic cell count; FCM: Fat corrected milk (3.5%); UDM: Useful dry matter content (fat + protein); TS: Total solids; NFTS: Non-fat total solids; abc: Least square means within a column having different superscripts differ significantly. * *p* < 0.05; ** *p* < 0.01; *** *p* < 0.001.

**Table 4:** Milk mineral profile according to the effects considered.

Mineral	Diets								
	C	AP25	AP40	AP60	BB25	BB40	BB60	SEM	Significance
Na (g/kg DM)	2.59	2.40	2.23	2.36	2.53	2.41	2.68	0.113	n.s.
Mg (g/kg DM)	0.888	0.837	0.835	0.932	0.884	0.813	0.853	0.047	n.s.
K (g/kg DM)	12.0	11.5	11.2	11.8	12.1	10.9	11.5	0.51	n.s.
Ca (g/kg DM)	8.85	7.56	8.64	8.81	8.07	7.85	7.81	0.495	n.s.
P (g/kg DM)	6.00	5.16	6.37	6.08	5.43	6.05	6.11	0.412	n.s.
S (g/kg DM)	2.45	2.29	2.44	2.45	2.35	2.40	2.37	0.107	n.s.
Se (mg/kg DM)	0.102	0.095	0.127	0.117	0.091	0.105	0.093	0.010	n.s.
Zn (mg/kg DM)	18.6	21.3	17.1	28.3	25.9	20.4	23.5	2.60	n.s.
Cu (mg/kg DM)	0.697	0.538	1.11	0.397	0.357	0.382	0.420	0.367	n.s.
Fe (mg/kg DM)	2.95	2.16	2.26	2.72	2.11	2.22	2.34	0.557	n.s.
Mn (mg/kg DM)	0.203 b	0.198 b	0.233 a	0.201 b	0.222 ab	0.185 b	0.192 b	0.010	*

C: Control diet; AP: Diet that includes artichoke plant silage; BB: Diet that includes broccoli by-product silage; 25, 40, and 60 inclusion level of by-product silage on dry matter basis %; SEM: Standard error mean. abc: Least square means within a column with different superscripts differ significantly. * *p* < 0.05.

**Table 5:** Fatty acid composition (g/100 g total fatty acids) measured in milk according to the effects considered.

Fatty Acid	Diets								
	C	AP25	AP40	AP60	BB25	BB40	BB60	SEM	Significance
C4:0	2.21	2.66	2.53	2.57	2.53	2.62	2.67	0.586	n.s.
C6:0	3.05	3.59	3.41	3.51	3.54	3.55	3.61	0.795	n.s.
C7:0	0.052 ab	0.060 ab	0.070 ab	0.046 b	0.073 ab	0.071 ab	0.077 a	0.024	*
C8:0	4.11	4.57	4.67	4.32	4.64	4.77	4.28	0.981	n.s.
C9:0	0.065 b	0.077 ab	0.095 a	0.088 ab	0.102 a	0.102 a	0.102 a	0.023	*
C10:0	13.2	15.0	14.7	14.5	15.6	15.6	15.3	3.03	n.s.
C10:1 c9	0.037	0.040	0.033	0.036	0.047	0.036	0.034	0.017	n.s.
C11:0	0.197 ab	0.171 bc	0.186 abc	0.157 c	0.190 ab	0.201 a	0.193 ab	0.022	**
C12:0	3.23 a	2.81 bc	3.10 abc	2.66 c	3.11 abc	3.31 ab	2.93 abc	0.274	***
C12:1 c9	0.032	0.024	0.035	0.030	0.039	0.037	0.024	0.012	n.s.
iso C13:0	0.017 b	0.016 b	0.026 ab	0.028 a	0.027 a	0.016 b	0.019 ab	0.008	*
anteiso C13:0	0.025	0.025	0.030	0.030	0.030	0.031	0.026	0.008	n.s.
iso C14:0	0.055 b	0.045 b	0.060 b	0.067 ab	0.063 ab	0.058 b	0.084 a	0.019	**
C14:0	7.62 ab	7.08 ab	6.92 ab	6.74 b	7.59 ab	7.56 ab	7.76 a	0.568	*
iso C15:0	0.174 abcd	0.130 b	0.178 abc	0.184 a	0.163 abcd	0.154 bc	0.152 bcd	0.021	***
anteiso C15:0	0.226 a	0.170 c	0.208 ab	0.223 a	0.189 bc	0.181 c	0.181 c	0.021	***
C14:1 c9	0.073 bc	0.062 c	0.067 bc	0.076 abc	0.071 bc	0.080 ab	0.090 a	0.011	***
C15:0	0.652 bc	0.524 d	0.617 c	0.753 ab	0.675 bc	0.717 b	0.818 a	0.066	***
C15:1	0.070 a	0.042 d	0.048 cd	0.064 ab	0.055 bc	0.061 ab	0.055 bcd	0.011	***
iso C16:0	0.176 c	0.147 d	0.188 bc	0.225 a	0.178 c	0.204 ab	0.218 a	0.022	***
C16:0	21.5 ab	22.3 ab	20.4 ab	20.5 b	22.1 ab	22.0 ab	23.9 a	1.67	**
C16:1 t4	0.039 ab	0.003 b	0.040 ab	0.070 a	0.003 b	0.024 ab	0.048 ab	0.049	*
C16:1 t5	0.023 ab	0.005 ab	0.029 ab	0.043 a	0.000 b	0.007 ab	0.042 ab	0.036	*
C16:1 t6-7	0.105	0.089	0.112	0.139	0.097	0.060	0.085	0.148	n.s.
C16:1 t9	0.193	0.168	0.187	0.166	0.188	0.175	0.137	0.114	n.s.
C16:1 t10	0.028	0.002	0.020	0.013	0.030	0.007	0.012	0.034	n.s.
C16:1 t11-12	0.012	0.041	0.023	0.048	0.019	0.063	0.041	0.037	n.s.
C16:1 c7	0.203	0.182	0.205	0.204	0.191	0.178	0.176	0.043	n.s.
C16:1 c9	0.436 c	0.449 bc	0.491 bc	0.542 ab	0.482 bc	0.475 bc	0.617 a	0.080	**
C16:1 c10	0.029 ab	0.000 b	0.031 ab	0.047 a	0.000 b	0.012 ab	0.033 ab	0.040	*
C16:1 c11	0.000	0.002	0.004	0.006	0.000	0.003	0.011	0.009	n.s.
iso C17:0	0.249 ab	0.234 ab	0.275 a	0.223 ab	0.207 ab	0.184 b	0.165 b	0.060	**
anteiso C17:0	0.287 a	0.218 bc	0.263 ab	0.293 a	0.257 ab	0.180 c	0.282 a	0.049	***
C17:0	0.555 b	0.485 b	0.516 b	0.703 a	0.536 b	0.541 b	0.636 a	0.058	***
C17:1 c6-7	0.040	0.046	0.050	0.049	0.041	0.056	0.034	0.018	n.s.
C17:1 c8	0.000 b	0.002 b	0.000 b	0.003 b	0.002 b	0.014 b	0.035 a	0.012	***
C17:1 c9	0.104 b	0.114 b	0.121 b	0.195 a	0.119 b	0.159 a	0.215 a	0.023	***
isoC18:0	0.034 ab	0.041 ab	0.047 b	0.047 ab	0.034 b	0.057 a	0.053 ab	0.013	*
C18:0	14.1 a	12.5 ab	13.2 ab	12.2 ab	12.7 a	11.8 ab	9.9 b	0.85	***
C18:1 t4	0.068 ab	0.085 a	0.067 ab	0.049 bc	0.082 a	0.043 c	0.045 c	0.016	***
C18:1 t5	0.030 ab	0.024 b	0.031 ab	0.033 ab	0.038 a	0.017 b	0.026 ab	0.011	**
C18:1 t6-8	0.196 a	0.166 abc	0.180 ab	0.134 d	0.146 bcd	0.171 abc	0.123 cd	0.027	**
C18:1 t9	0.269 a	0.271 ab	0.245 abc	0.234 bcd	0.233 bcd	0.213 abcd	0.193 d	0.028	**
C18:1 t10	0.276 a	0.235 ab	0.230 ab	0.205 b	0.220 ab	0.235 ab	0.219 b	0.047	*
C18:1 t11	1.30 a	1.33 a	1.35 a	1.25 ab	0.98 bc	0.95 c	0.81 c	0.169	***
C18:1 t12	0.492 a	0.471 a	0.460 abc	0.396 b	0.383 bcd	0.377 bcd	0.317 d	0.049	***
C18:1 t13-14	0.059	0.000	0.058	0.000	0.062	0.114	0.037	0.117	n.s.
C18:1 c9	18.0 ab	17.6 ab	18.2 ab	19.0 a	16.3 b	16.9 ab	175 ab	1.45	*
C18:1 c11	0.043 ab	0.055 ab	0.038 ab	0.005 b	0.045 ab	0.155 a	0.052 ab	0.121	*
C18:1 c12	0.587 a	0.565 abc	0.581 a	0.536 abc	0.511 bc	0.569 ab	0.511c	0.047	**
C18:1 c13	0.124	0.116	0.112	0.115	0.115	0.119	0.112	0.019	n.s.
C18:1 c14	0.424 a	0.395 ab	0.375 ab	0.326 b	0.371 b	0.365 b	0.329 b	0.040	**
C18:1 c15	0.206	0.192	0.195	0.213	0.198	0.208	0.209	0.028	n.s.
C18:2 c9,t13	0.294 a	0.229 abc	0.246 ab	0.188 c	0.220 bc	0.220 abc	0.174 abc	0.044	**
C18:2 t8,c13	0.098 a	0.084 ab	0.083 ab	0.089 ab	0.074 b	0.089 ab	0.092 ab	0.019	*
C18:2 t9,12	0.000	0.000	0.007	0.057	0.000	0.000	0.008	0.034	n.s.
C18:2 c9,t12	0.154 a	0.117 ab	0.112 b	0.104 b	0.106 b	0.107 b	0.101 b	0.031	**
C18:2 t11,c15	0.011 ab	0.004 b	0.014 a	0.017 a	0.013 ab	0.010 b	0.017 a	0.008	**
C18:2n6	2.59 abcd	2.40 ab	2.42 ab	2.53 a	2.10 c	2.26 bc	1.98 bcd	0.193	***
C20:0	0.233 d	0.267 bc	0.280 b	0.350 a	0.237 cd	0.241 cd	0.225 d	0.029	***
C18:3n6	0.025	0.022	0.027	0.023	0.015	0.010	0.019	0.014	n.s.
C20:1 c9	0.012 ab	0.010 b	0.017 ab	0.029 a	0.000 b	0.007 b	0.008 b	0.015	**
C20:1 c11	0.038	0.050	0.053	0.049	0.052	0.053	0.040	0.018	n.s.
C18:3n3	0.183 b	0.145 c	0.152 bc	0.242 a	0.156 bc	0.179 bc	0.173 bc	0.025	***
CLA c9,t11	0.486 bc	0.510 abc	0.527 ab	0.538 ab	0.370 bc	0.377 c	0.344 bc	0.064	**
CLA t9,c11	0.044 b	0.032 c	0.038 bc	0.058 a	0.030 c	0.032 c	0.035 bc	0.009	***
CLA t10,c12	0.024	0.026	0.029	0.039	0.013	0.010	0.024	0.024	n.s.
CLA t12,14	0.017	0.012	0.023	0.025	0.009	0.006	0.022	0.017	n.s.
∑CLA	0.528 a	0.550 a	0.549 a	0.532 a	0.529 a	0.531 a	0.482 b	0.019	***
C20:2n6	0.033	0.027	0.038	0.040	0.044	0.036	0.034	0.015	n.s.
C20:3n9	0.070 b	0.061 b	0.075 b	0.116 a	0.080 b	0.060 b	0.069 b	0.017	***
C22:0	0.023	0.027	0.019	0.025	0.018	0.021	0.027	0.015	n.s.
C20:3n3	0.000 b	0.004 b	0.013 b	0.031 a	0.000 b	0.000 b	0.000 b	0.012	***
C20:4n6	0.152 a	0.126 b	0.151 a	0.165 a	0.158 a	0.146 ab	0.153 a	0.018	***
C23:0	0.021 bc	0.019 c	0.030 abc	0.047 a	0.045 a	0.029 abc	0.038 ab	0.015	**
C20:4n3	0.001	0.001	0.001	0.001	0.010	0.001	0.001	0.009	n.s.
C22:2n6	0.000 c	0.026 b	0.001 c	0.009 bc	0.051 a	0.023 b	0.057 a	0.015	***
C24:0	0.049	0.031	0.047	0.073	0.126	0.036	0.042	0.092	n.s.

C: Control diet; AP: Diet that includes artichoke plant silage; BB: Diet that includes broccoli by-product silage; 25, 40, and 60 inclusion level of by-product silage on dry matter basis %; SEM: Standard error mean; abc: Least square means within a column having different superscripts differ significantly. * *p* < 0.05; ** *p* < 0.01; *** *p* < 0.001.

**Table 6:** Grouped fatty acids (g/100 g total fatty acids) and indices related to cardiovascular health and desaturation activity in milk according to the effects considered.

Variable	Diets								
	C	AP25	AP40	AP60	BB25	BB40	BB60	SEM	Significance
SFA	72.2	73.0	72.2	70.9	75.1	74.2	73.6	2.19	n.s.
MUFA	23.3	22.7	23.5	24.5	21.1	21.8	22.6	1.90	n.s.
PUFA	4.11 ab	3.86 abc	3.87 abc	4.24 a	3.40 d	3.56 cd	3.50 bcd	0.335	***
UFA	27.4	26.6	27.4	28.7	24.5	25.4	26.1	2.21	n.s.
SFA/UFA	2.64	2.77	2.64	2.50	3.10	2.95	2.85	0.326	n.s.
SCFA	22.9	26.1	25.7	24.7	26.6	26.9	25.7	5.38	n.s.
MCFA	36.2 b	35.6 b	34.3 b	34.8 b	36.5 b	36.6 b	39.4 a	2.79	*
LCFA	39.8 abc	37.4 abc	38.7 abc	41.6 ab	36.4 abc	35.4 bc	36.0 c	2.88	**
n3	0.182 b	0.151 b	0.164 b	0.275 a	0.157 b	0.178 b	0.174 b	0.034	***
n6	2.78 a	2.55 abc	2.60 ab	2.79 a	2.30 c	2.44 bc	2.18 bc	0.218	***
n6/n3	15.4 abc	17.3 ab	17.4 a	10.3 d	14.8 abc	13.8 bc	12.3 cd	2.33	***
AI	2.11 b	2.11 bc	1.95 cd	1.83 d	2.37 a	2.28 ab	2.31 ab	0.127	***
TI	3.32 b	3.30 b	3.09 cd	2.94 d	3.65 a	3.39 b	3.36 abc	0.141	***
DI C14:0	0.010 abc	0.009 abc	0.010 abc	0.011 c	0.009 abc	0.011 bc	0.012 a	0.001	***
DI C16:0	0.050 b	0.044 b	0.055 ab	0.061 a	0.044 b	0.048 b	0.050 ab	0.009	**
DI C18:0	1.55 bc	1.72 bc	1.67 b	1.80 ab	1.54 d	1.75 bc	2.08 a	0.049	***

C: Control diet; AP: Diet that includes artichoke plant silage; BB: Diet that includes broccoli by-product silage; 25, 40, and 60 inclusion level of by-product silage on dry matter basis %; SEM: Standard error mean; SFA: Saturated fatty acids; MUFA: Monounsaturated fatty acids; PUFA: Polyunsaturated fatty acids; UFA: Unsaturated fatty acids (MUFA + PUFA); SCFA: Short chain fatty acids (C6:0 a C10:0); MCFA: Medium chain fatty acids (C11:0 a C17:0); LCFA: Long chain fatty acids (C18:0 a C24:0); AI: Atherogenic index; TI: Thrombogenic index; DI: Desaturation index; abc: Least square means within a column having different superscripts differ significantly. * *p* < 0.05; ** *p* < 0.01; *** *p* < 0.001.

**Table 7:** Plasmatic profile according to the effects considered.

Variable	Diets								Significance		
	C	AP25	AP40	AP60	BB25	BB40	BB60	SEM	Diet	Sampling	Diet x Sampling
Glucose (mg/dL)	44.6 bc	47.7 ab	45.0 bc	48.3 ab	42.5 c	49.5 a	50.0 a	1.52	***	***	***
Plasma urea (mg/dL)	52.0 a	50.7 a	50.9 a	49.2 a	38.8 bc	39.8 b	33.2 c	2.14	***	**	***
BHB (mmol/L)	0.336 bc	0.522 a	0.424 ab	0.376 bc	0.421 ab	0.299 c	0.304 c	0.040	***	n.s.	**

C: Control diet; AP: Diet that includes artichoke plant silage; BB: Diet that includes broccoli by-product silage; 25, 40, and 60 inclusion level of by-product silage on dry matter basis %; SEM: Standard error mean; BHB: β-hydroxybutyrate; abc: Least square means within a column having different superscripts differ significantly. ** *p* < 0.01; *** *p* < 0.001.
